# Experimental Study on the Relationship between the Velocity of Surface Movements and Tilting Rate in Pre-Failure Stage of Rainfall-Induced Landslides

**DOI:** 10.3390/s21185988

**Published:** 2021-09-07

**Authors:** Jiren Xie, Taro Uchimura, Chao Huang, Zain Maqsood, Jingli Tian

**Affiliations:** 1 Institute of Geographic Sciences and Natural Resources Research, Chinese Academy of Sciences, Beijing 100101, China; jirenxie198911@csu.edu.cn; 2 Department of Civil Engineering, Central South University, Changsha 410075, China; tianjingli@csu.edu.cn; 3 Department of Civil and Environmental Engineering, Saitama University, 255 Shimo-Okubo, Sakura-ku, Saitama 338-8570, Japan; uchimurataro@mail.saitama-u.ac.jp; 4 Disaster Prevention Research Institute, Kyoto University, Uji, Kyoto 611-0011, Japan; 5 School of Civil and Environmental Engineering, National University of Sciences and Technology, H-12, Islamabad 44000, Pakistan; zmaqsood@nice.nust.edu.pk

**Keywords:** rainfall-induced landslides, tilting rate, velocity of surface displacements, laboratory tests and field tests, linear relationship, prediction

## Abstract

With the development of deformation measuring technology at slope surfaces, prediction methods for rainfall-induced landslides based on the surface movements and tilting of slopes in the pre-failure stage have been recognized as a promising technique for risk reduction of slope failure triggered by rainfall. However, the correlation and possible mechanism for these prediction methods were rarely discussed. In this study, the comparison between the prediction methods of slope failure based on the time history of surface displacements and tilting in the acceleration stage was carried out by conducting a series of laboratory tests and field tests under rainfall, in which the movements and tilting behaviors at the slope surface were measured. The results show that the predicted failure time of tested slopes obtained by different prediction methods is consistent, and the correlation between these landslide prediction methods were also detected. A proportional relationship between the velocity of surface displacements and tilting rate was observed, and a possible mechanism for the sliding behavior was discussed based on this linear relationship as well. In addition, an expression for the linear relationship between the rate of the surface tilting and displacement was also established in this study, and the results could have significance for the understanding of the sliding behavior in the failure process in rainfall-induced landslides.

## 1. Introduction

Rainfall-induced landsides often lead to serious damage on environment, infrastructures, and human lives. It was reported that the rainfall-induced landslides cause thousands of fatalities and billions of dollars in economic losses every year [1,2,3,4,5]. 

Longstanding effort has been made to mitigate the risk of damages caused by rainfall-induced landslides, which was recognized as a difficult task due to many influential factors, such as rainfall, geotechnical properties of soils on slopes, or the ground water level and so on. Traditional methods for landslide damage reduction, such as retaining walls and piles, improve the factor of safety by mechanical reinforcement measures. These methods are costly and not suitable for a large number of slopes with potential risks of failure. 

In recent decades, early warning methods of rainfall-induced landslides with low cost and negligible environmental impact have been proposed and recognized as promising approaches for the risk reduction of landslide disasters. These methods are used to detect the pre-failure behavior of landslides triggered by rainfall and evacuate the residents living in the landslide prone in a timely manner [6,7,8,9,10]. Centrifuge and mechanics-based models were used to study and assess rainfall-induced instabilities in different slopes [11,12], and climate change increasing the amount of antecedent rainfall significantly affects the rainfall-induced landslide vulnerability of the area [13].

Many landslide prediction methods were proposed based on the soil moisture measurement [14,15] and local rainfall record [16,17,18]. These methods are not effective to evaluate the risk of the slope failure due to the unclear correlation between the soil moisture content or precipitation and the occurrence of landslides. The typical landslide prediction methods were derived from the surface displacement, and many empirical equations using derivatives of surface displacements were also developed to estimate the slope failure time [19,20,21]. A general form of these empirical equations were firstly given by Fukuzono expressed as Equation (1) [22,23]. However, the mechanism of these typical landslide prediction methods is still unclear.

(1) $$\frac{dt}{ds}={\left[A\cdot (\alpha -1)\right]}^{\frac{1}{\alpha -1}}{\left(t_{f}-t\right)}^{\frac{1}{\alpha -1}}$$

where $\frac{dt}{ds}$ denotes reciprocal displacement rate. A and α are constant parameters. t represents the time, and $t_{f}$ means the slope failure time at which the displacement rate becomes infinitely large. It was reported that the value of α is close to 2 [23,24,25,26,27], so the above equation can be rewritten as

(2) $$\frac{dt}{ds}=A\times (t_{f}-t)$$

Additionally, a new landslide prediction method based on the surface tilting was proposed by Xie [28]. This method adopts the following equation to forecast the slope failure time

(3) $$\frac{dt}{\left|d\theta \right|}=\frac{1}{B}\times (t_{F}-t)$$

where $\frac{dt}{\left|d\theta \right|}$ is the inverse number of the tilting rate, and B is a constant parameter, while t means the time and $t_{F}$ represents the slope failure time when the reciprocal tilting rate becomes 0 min/°. As shown in Equations (2) and (3), the formula of landslide predicting methods based on the time history of surface displacements and tilting are analogue, which indicate that the reciprocal velocity of surface deformation including displacements and tilt angles reveals a linear relationship with the duration before the slope failure. However, limited studies were carried to investigate the mechanism of the similarity between these methods, and the correlation between the deformation indexes used in different prediction methods. 

In this paper, a comparison between the landslide prediction methods using the surface tilting and displacements was studied by performing a series of model tests at both laboratory and field scales, in which extensometers and tilt sensors were used for the measurement of surface movements and tilt angles. The relationship between the tilting rate and displacement rate was also investigated and validated.

## 2. Methods and Materials

To investigate the correlation between the landslide predicting methods based on the surface tilting and displacements of slopes, a series of laboratory model tests and field tests were carried out. 

### 2.1. Laboratory Model Tests

**Figure 1:**
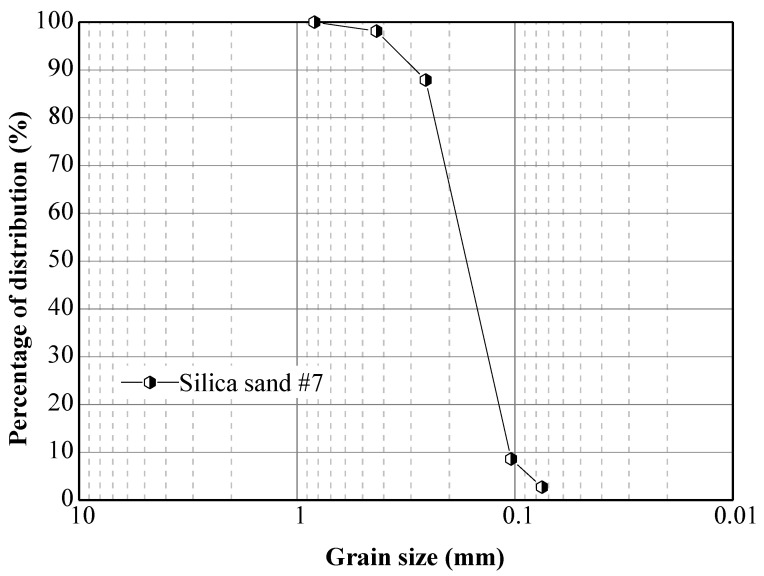
Particle size distribution of silica sand number 7.

Two small flume tests were performed. In Model Test 1, the slope model was made in a rectangular container with the size of 1165 mm (length) × 450 mm (width) × 380 mm (height), and composed of two layers with different dry density using silica sand number 7. The particle size distribution of the materials is presented in Figure 1, and the specific gravity of soil solids (G_s_) is 2.63. The dry density of the base layer is 1.60 g/cm^3^ with the void ratio of 0.64, while the dry density of the surface layer is 1.32 g/cm^3^, of which the void ration is around 1.

**Figure 2:**
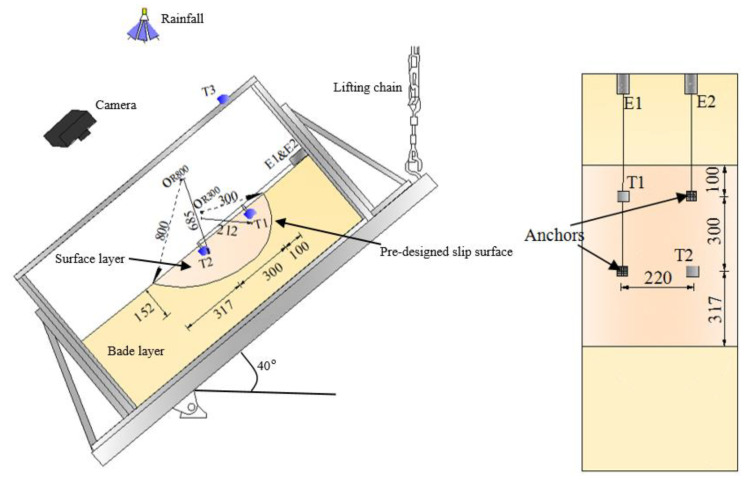
Illustration of the arrangement of instruments in Model Test 1.

The slope model composed of two different layers with specified densities was built by tamping, and the initial moisture content is 10%. A pre-designed irregular circular slip surface with different radiuses was set between these two layers as shown in Figure 2. The radius of the slip surface of the upper part was 300 mm, and it was 800 mm in the lower part. After making the slope model at horizontal position, this slope model was inclined to a target angle about 40 degrees before artificial rainfall with the rainfall intensity of 70 mm/h applied. The intensity of artificial rainfall was controlled by a rainfall system, which was composed of a nozzle, a water tank and an air pressure cylinder. Two tilt sensors and extensometers which are noted by “T” and “E” were exploited in this test with the accuracy of 0.1 degree and 0.1 mm to measure the pre-failure tilting behavior and the surface displacement of the slope as indicated in Figure 2. 

**Figure 3:**
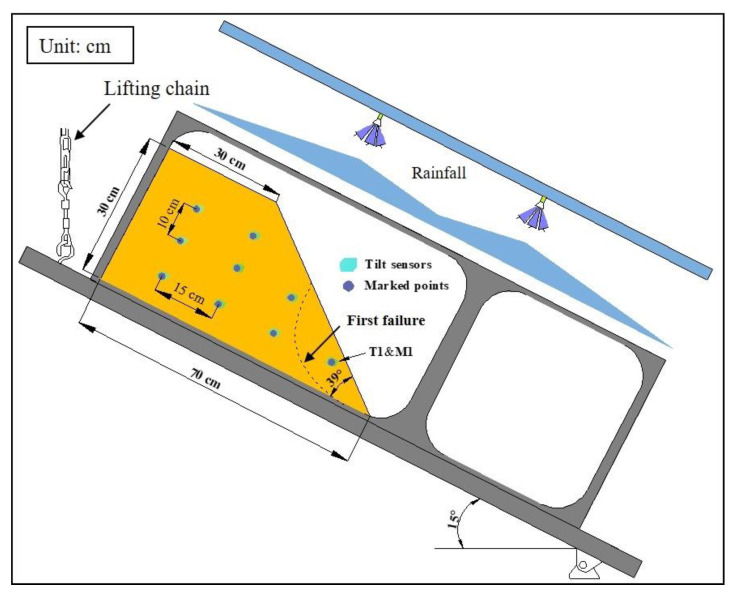
Illustration of the arrangement of instruments in Model Test 2.

In Model Test 2, the slope model was also made of silica sand number 7, and the dry density of the slope is 1.32 g/cm^3^ with an initial moisture of 10%. The slope dimension of this model is shown in Figure 3. Nine marked points using wooden sticks of 5 cm were set in the slope close to the location where tilt sensors were installed. A digital camera parallel to the slope profile was used to record the movement of marked points in the slope based on the image technique, while the slope tilting behavior was measured by the tilt sensors. The slope model in this test was also built horizontally, and then inclined gradually to a specific angle of 15 degree using the lifting mechanism as shown in Figure 3 before the artificial rainfall with a constant intensity of 50 mm/h applied. In this test, nine tilt sensors with the accuracy of 0.1 degree were used, and behaviors in the first slope failure occurred at the toe of the slope was captured by the tilt sensor 1 (T1) and marked point 1 (M1).

### 2.2. Field Tests

Two field tests were performed on natural slopes in Baise city of Guangxi province where the weakly expansive clay is widely distributed. The chosen site, located in western–northwestern Guangxi, has a humid subtropical climate. Rainfall is relatively low compared to eastern locations in Guangxi, averaging around 1000 mm per annum, a majority of which occurs from June to August. The western parts of location, with an average elevation surpassing 500 m, lie along the southeastern fringes of the Yunnan–Guizhou Plateau. The area is the prone and frequent occurring areas of geological hazards including landslides and debris flow. 

**Figure 4:**
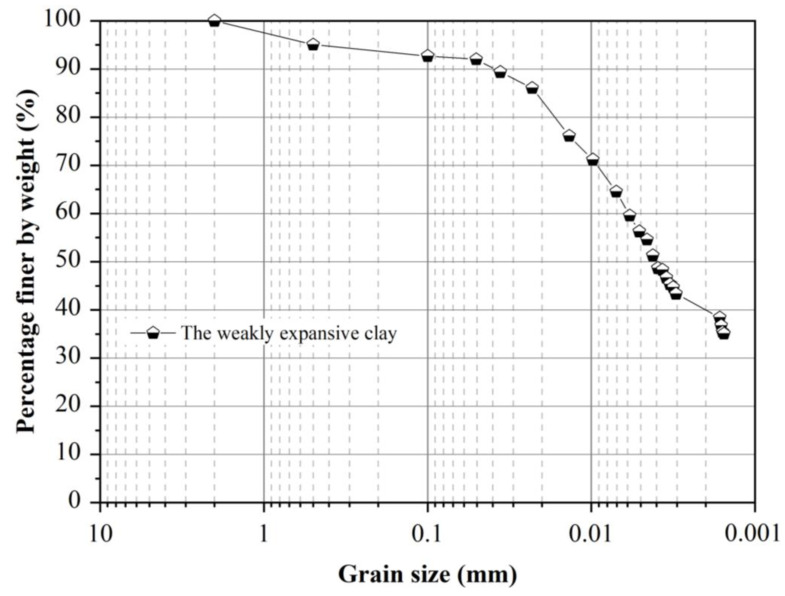
Particle size distribution of the soil in field tests.

**Figure 5:**
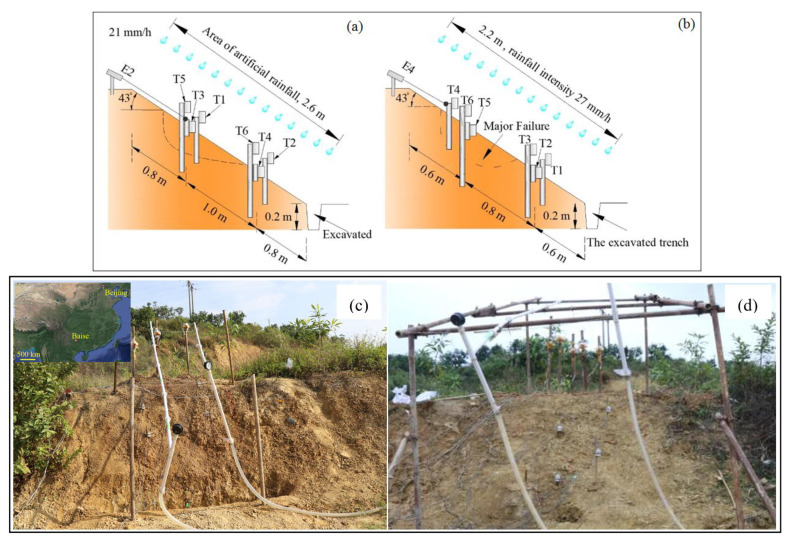

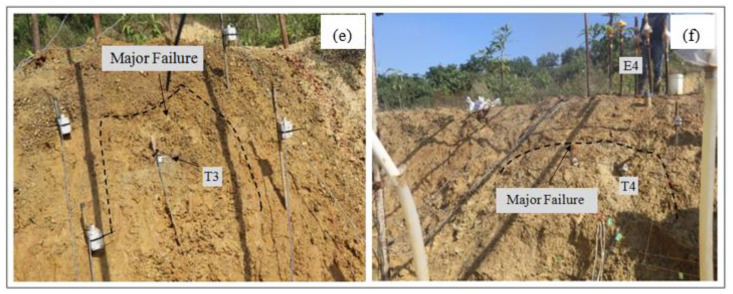
(**a**) The arrangement of instruments in Field Test 1; (**b**) The arrangement of instruments in Field Test 2; (**c**) The test slope in field test 1; (**d**) The test slope in Field Test 2; (**e**) The major failed part of the test slope in Field Test 1; (**f**) The major failed part of the test slope in Field Test 2. Tilt sensors and extensometers which are noted by “T” and “E” were exploited in this test.

Particle size distribution of the in-situ soil is presented in Figure 4. The angle of these test slopes was around 40 degrees, and a trench was excavated at the toe of the slope with a depth of 0.2 m to make the slope easier to collapse. In Field Test 1, six tilt sensors and three extensometers were exploited to monitor the slope surface deformation, and the experimental setup is indicated in Figure 5a,c. The three extensometers were attached to the tilt sensors T1, T2, and T3 which were located at the bottom area. After the installation of tilt sensors, artificial rainfall was applied with a constant rainfall intensity of 21 mm/h using the rainfall supply system which was composed of six nozzles, a pump, and several tubes. 

The major failure occurred in the middle part of the slope four hours later as shown in Figure 5e. Compared with the test conditions in Field Test 1, six extensometers together with tilt sensors were used in this field test, and a slight different rainfall intensity of 27 mm/h was applied in Field Test 2. The arrangement of apparatus in the test as shown in Figure 5b,d. Similarly, the major failure was also occurred in the middle part of the slope revealed in Figure 5f, and the pre-failure behavior of the slope surface was recorded by the extensometer, E4 as well as the tilt sensor, T4.

## 3. Test Results and Discussion

### 3.1. Results of Model Test 1

**Figure 6:**
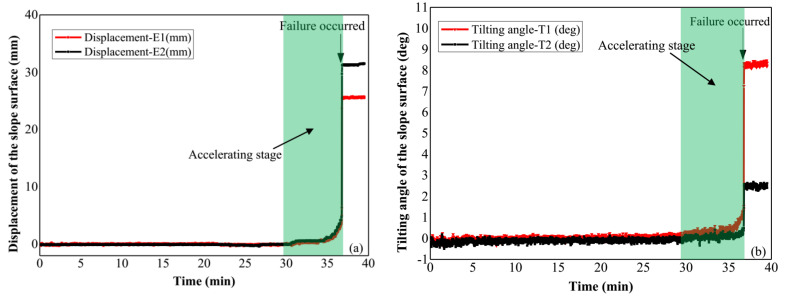
(**a**) Time history of the displacement at the slope surface in Model Test 1, (**b**) time series of the tilting angle at the slope surface.

In this test, the slope failure was triggered by applying artificial rainfall with constant rainfall intensity of 70 mm/h, and the slope slid along the pre-designed slip surface. The pre-failure tilting behavior and displacement of the slope surface were detected by the tilt sensors and extensometers as shown in Figure 2. Time series of surface displacements and tilting angles measured by the extensometers and tilt sensors are presented in Figure 6a,b respectively. An accelerating stage of surface deformation before the slope failure is shown in these figures. 

**Figure 7:**
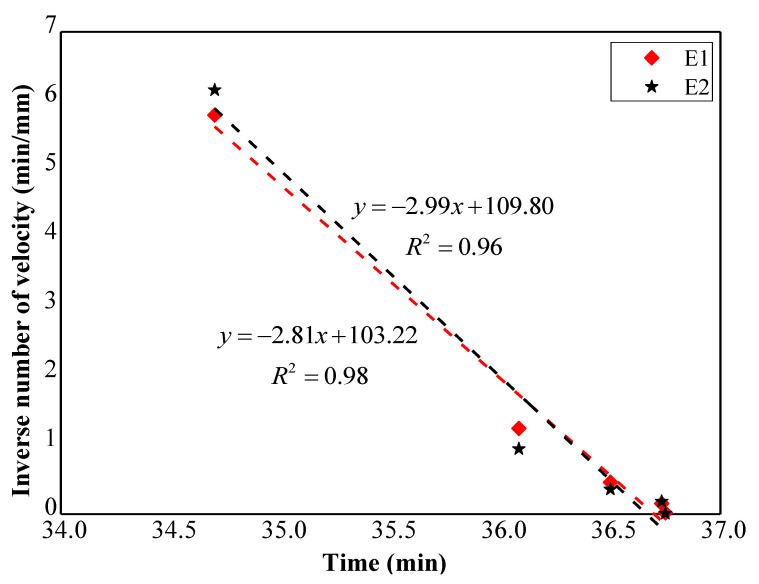
Time series of the reciprocal displacement rate of E1 and E2.

**Figure 8:**
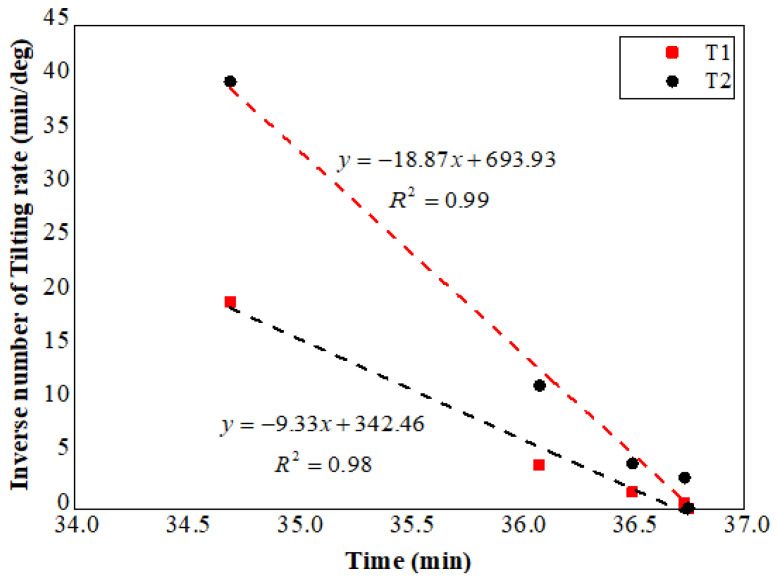
Time series of the reciprocal tilting rate of T1 and T2.

In addition, the relationship between reciprocal rates of surface deformation and the time is presented in Figure 7 and Figure 8, which is based on the time history of surface displacement and tilting respectively. The method for the calculation of the reciprocal rate of surface deformation is provided in Appendix A of this paper. The failure time of the slope forecasted using Equation (2) based on the linear trends revealed in Figure 7 is close to 36.7 min. Moreover, the predicted slope failure time was obtained using Equation (3) according to the relationship between the reciprocal tilting rate and time indicated in Figure 8, which also approximates to the real failure time, 36.7 min. The results reveal that consistent predicted failure time of slopes can be achieved using the typical landslide forecasting method based on the surface displacement and the new prediction method proposed by authors derived from the slope surface tilting.

### 3.2. Results of Model Test 2

**Figure 9:**
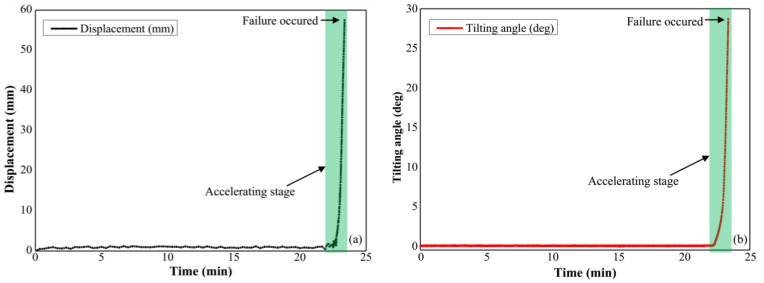
(**a**) Time history of the displacement at subsurface in Model Test 2, (**b**) time series of the tilting angle at the subsurface in Model Test 2.

Compared with Model Test 1, the slope failure of this test was also induced by applying artificial rainfall with a constant rainfall intensity, which was 50 mm/h. The slope failure began at the toe of the slope before the second failure occurred. The displacement of the slope in the first failure presented in Figure 9a was derived from the movement of the marked point set in the failed part using the image analysis technique. Corresponding tilting behaviors of the failure part was recorded by the tilt sensor located in this region is presented in Figure 9b. An accelerating stage of the surface deformation including displacements and tilting angles before the slope failure is revealed in time history of the surface displacement and tilting. 

**Figure 10:**
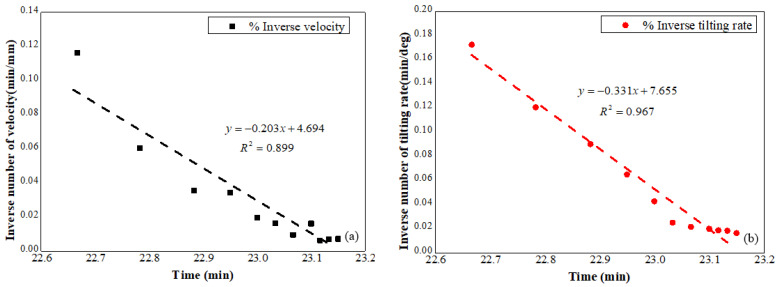
(**a**) Reciprocal displacement rate against time, (**b**) reciprocal tilting rate against time.

The correlation between inverse number of displacement rate and time in the acceleration stage is presented in indicated in Figure 10a, while the relationship between the reciprocal tilting rates and time is shown in Figure 10b. Linear relations between the reciprocal rate of displacements and tilting angles against time are presented in Figure 10a,b, and the fitting lines for these linear relations are also presented in these figures. The predicted slope failure time was computed using Equations (2) and (3) based on the fitting lines as shown in Figure 10, and a consistent result was obtained, which is 23.12 min approximating to the real failure time.

### 3.3. Results of Field Test 1

**Figure 11:**
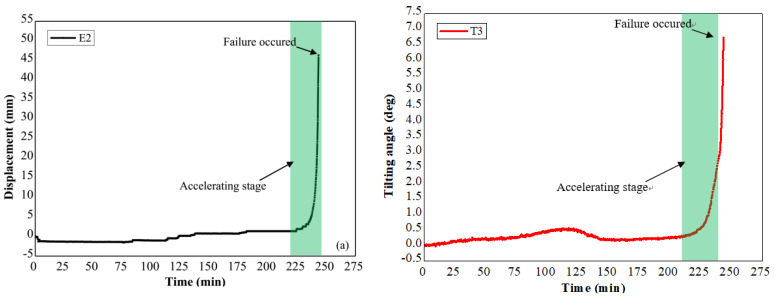
(**a**) Time history of the displacement at subsurface in Field Test 1, (**b**) time series of the tilting angle at the subsurface in Field Test 1.

The slope failure in Field Test 1 was caused by applying artificial rainfall with the rainfall intensity of 21 mm/h. The major failure occurred in the middle part of the slope where tilt sensor T3 and the extensometer E2 located. The failed part slid along the slip surface with a depth of 23 cm, and the cumulative displacement and tilting angle measured by E2 and T3 are provided in Figure 11. A similar trend between the surface displacement and tilting angle against time is indicated in these figures, and the accelerating behavior is also observed both in the time history of surface displacements and tilting angles before the slope failure.

**Figure 12:**
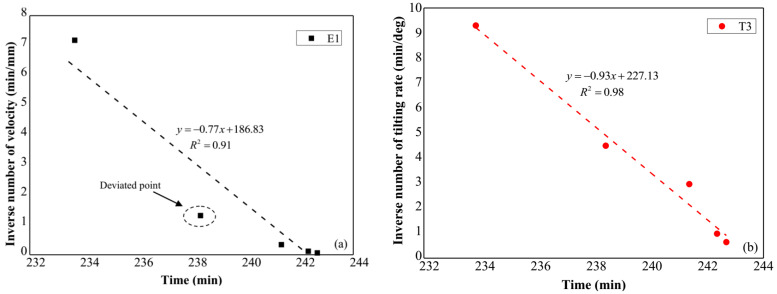
(**a**) Reciprocal displacement rate against time, (**b**) reciprocal tilting rate against time.

Figure 12a shows the relation between the reciprocal displacement rate and time, which was obtained using the surface displacement measured by E2, while the time series of the reciprocal tilting rate is indicated in Figure 12b. Linear trends are implied in these two figures, and the slope failure time forecasted based on these trends using Equation (2) and Equation (3) is 242.6 min and 244.2 min respectively. The slight difference for the predicted failure time of the slope is caused by the variation of the data as shown in Figure 12a.

### 3.4. Results of Field Test 2

**Figure 13:**
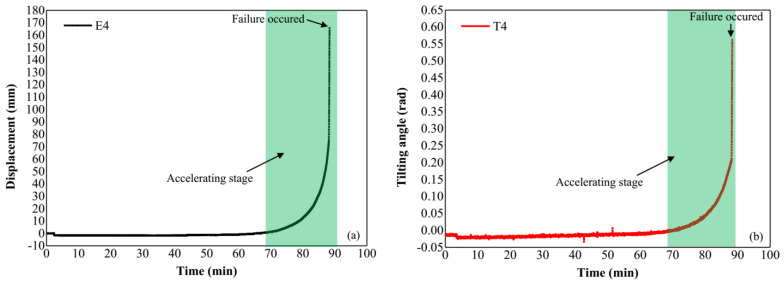
(**a**) Time history of the displacement at subsurface in Field Test 2, (**b**) time series of the tilting angle at the subsurface in Field Test 2.

The site of Field Test 2 is also located in Baise city, and the test conditions are similar as that in Field Test 1 with a different rainfall intensity of 27 mm/h. The failure occurred in the middle part of this slope, and the pre-failure behavior was recorded by the extensometer E4 and the tilt sensor T4 installed in this region as presented in Figure 5. The tilting angle and displacement of the failed part are presented in Figure 13a,b, respectively. As shown in Figure 13, the surface displacement and tilting increased sharply at the vicinity of 68 min until the slope failure occurred. A clear accelerating stage is also revealed in the time history of surface displacements and that of surface tilting, coinciding with the result indicated in other tests. 

**Figure 14:**
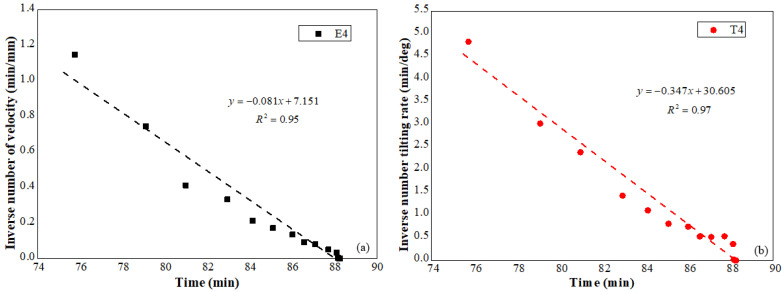
(**a**) Reciprocal displacement rate against time, (**b**) reciprocal tilting rate against time.

The relationship between the inverse number of the displacement and tilting rate against time in the acceleration stage is implied in Figure 14a,b respectively. Linear relations between rates of surface deformation against time are indicated in these figures, and the predicted slope failure derived from the linear trends is 88.28 min and 88.20 min receptively, consistent with the actual slope failure time.

## 4. Discussion

### 4.1. A Comparison between Landslide Prediction Methods Based on Surface Displacements and Tilting

Results of the laboratory and field tests indicate that the surface tilting increases synchronously with the increment of the displacement at slope surfaces. Additionally, the reciprocal rate of displacement and surface tilting against time were also presented in this study, and the first comparison between the landslide prediction method using surface tilting and that based on the time history of the surface displacement was carried out. In Model Test 1, failure of the slope with a pre-defined slip surface was triggered by rainfall infiltration. Consistent predicted failure time of the slope was achieved using the different landslide prediction method based on the tilting behavior and displacement of slope surface respectively. This result reveals that the landslide prediction methods based on the index of surface deformation are applicable for the slope with a pre-existed slip surface. 

Compared with the test conditions in Model Test 1, a homogeneous slope model made of silica sand was exploited in Model Test 2 to investigate the reliability of the predicted slope failure time evaluated by Equations (2) and (3). The result in this test also indicate that the failure time of the slope estimated by the landslide forecasting method based on the surface displacement and tilting using Equations (2) and (3) are consistent, which are in good agreement with the real failure time of this slope. 

Additionally, two field tests with varying rainfall intensity were also performed. As shown in Figure 11 and Figure 13, accelerating stages of the surface displacement and tilting are indicated, coinciding with the results presented in the laboratory tests. Linear relationships between the rate of surface displacement and tilting against time are also revealed in Figure 12 and Figure 14. Similar as the results indicated in the laboratory tests, the predicted slope failure time estimated by Equations (2) and (3) based on the linear relations between the rate of surface deformation and time is consistent with the real failure time. 

Conclusively, either the traditional landslide prediction methods using the time history of surface displacement in the acceleration stage or the forecasting method of slope failure derived from the pre-failure slope surface tilting behavior can be used to obtain the slope failure time with high reliability, and differences causing by usage of these varying landslide prediction methods on the predicted failure time of slopes are negligible based on the results of laboratory tests and field tests.

### 4.2. Possible Relationship between the Rate of Surface Displacement and Tilting

The velocity of surface movements and the surface tilting rate have been considered as the significant indicator to assess the slope stability by many researchers in recent decades [26,27,28], and analogous landslide prediction methods using these indexes were also proposed as indicated in Equations (2) and (3). However, limited studies have been carried out to investigate relations between the velocity and tilting rate of the slope surface. In this study, the correlation between these indicators was explored and a possible mechanism for the slope failure was also discussed based on this correlation.

**Figure 15:**
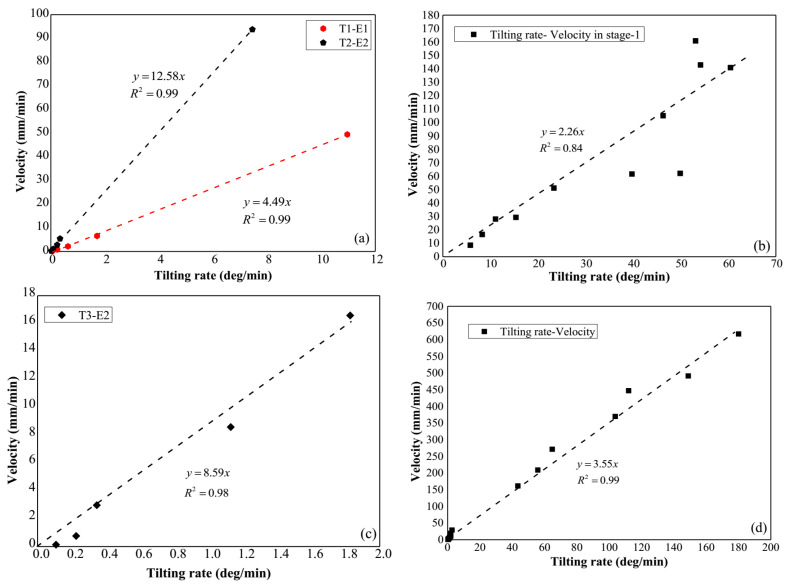
(**a**) Relationship between the tilting rate and the displacement rate in Model Test 1, (**b**) relationship between the tilting rate and the displacement rate in Model Test 2, (**c**) relationship between the tilting rate and the displacement rate in Field Test 1, (**d**) relationship between the tilting rate and the displacement rate in Field Test 2.

As shown in Figure 15, the correlation between the tilting rate and velocity of surface movements in the laboratory tests and field tests of this study is revealed. Linear relations between these two indicators are indicated with high coefficients of determination (R^2^). As a result, a possible expression for the relation between the rate of surface displacements and tilting is given as

(4) $$\frac{ds}{dt}=C\times \frac{d\theta }{dt}$$

where C is a coefficient of proportionality. $\frac{ds}{dt}$ and $\frac{d\theta }{dt}$ represent for the displacement rate and tilting rate, respectively.

In addition, it is obvious that if the inverse number of velocity in Equation (2) is substituted by Equation (4), the Equation (3) based on the surface tilting behavior can be achieved. This result provides the evidence to explain the similarity between Equations (2) and (3), and also gives the verification for the consistent estimated slope failure time obtained by different prediction methods as mentioned before.

**Figure 16:**
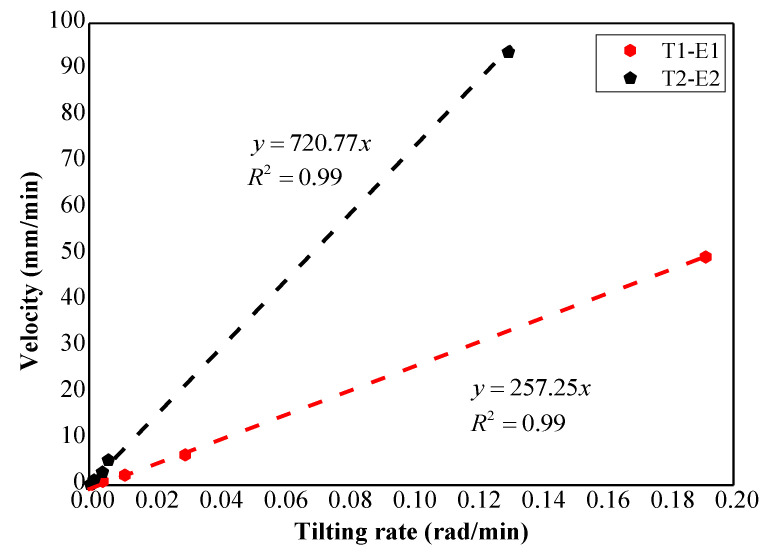
Tilting rate with unit rad/mm against velocity in Model Test 1.

Furthermore, the relations between the tilting rate and displacement rate in Model Test 1 with a pre-designed slip surface also reveal that the coefficients of these relations, which are 257.25 and 720.77 when the rate of surface displacements plot against the tilting rate using the unit radians instead of degrees as shown in Figure 16, approximate to the actual distances between centers of the slip surface and the location tilt sensors were installed with the values of 212 mm and 685 mm respectively. 

This result implies that the deformation at the slope surface is mainly caused by the rotation of the sliding masses along the slip surface, and the internal deformation of the sliding mass is negligible. Considering this, we can infer that the possible mechanism for slope surface deformation in acceleration stage before slope failure is controlled by the reduction of shear resistance of the slip surface. Additionally, the result also indicates that the coefficient presented in Equation (4), C, is related to the geometry of the slip surface.

## 5. Conclusions

In this study, the first comparison between the landslide predicting methods based on the surface displacement and tilting was carried out by performing a series of the laboratory tests and a field test. In addition, the relationship between the tilting rate and displacement rate was also investigated. The major findings of this research can be drawn as follows:

1. The results of laboratory and field tests indicate that either the inverse velocity forecasting method or the landslide predicting method using the slope tilting measurement can be applied to evaluate the slope failure time, and the predicted failure time calculated by these two forecasting methods is consistent with the actual slope failure time.
2. Linear relations between the tilting rate and displacement rate was observed, and an expression for the linear relations was also proposed, in which the coefficient, C, is found to be controlled by the geometry of slope slip surfaces.
3. The relationship or similarity between the landslide prediction methods derived from the history of surface displacements and tilting as shown in Equations (2) and (3) was elucidated based on the test results.
4. Based on the results in Model Test 1, a possible mechanism for slope surface deformation was proposed, in which the acceleration stage of surface deformation is induced by the reduction of shear resistance along the slip surface.

## Data Availability

Not applicable.

## Appendix A

The reciprocal rate of the displacement or tilting angle can be computed by the following equation,

(A1) $${\left(\frac{dt}{d\Omega }\right)}_{i+1}=\frac{t_{i+1}-t_{i}}{\Omega_{i+1}-\Omega_{i}}\;(i=0,\;1,\;\ldots ,\;n-1)$$

where Ω represents the displacement or tilting angle, and $\Omega_{i+1}$ is the value of the variables at the time $t_{i+1}$. ${\left(\frac{dt}{d\Omega }\right)}_{i+1}$ indicates the reciprocal rate of the variables at time $t_{i+1}$, and n is the dimension of the data sets.
